# Supplementary material for: Synthesis of New Indanyl Nucleoside Analogues and their Biological Evaluation on Hepatitis C Virus (HCV) Replicon
Source: Molecules. 2019 Mar 11;24(5):990. doi: 10.3390/molecules24050990 (PMC6429379; doi:10.3390/molecules24050990)
Supplement: Supplementary file 1 [file molecules-24-00990-s001.pdf]

Supplementary Materials

Synthesis of New Indanyl Nucleoside Analogues and their Biological Evaluation on Hepatitis C Virus (HCV) Replicon

Matías E. Gómez [1,†], Emiliano A. Gentile [2,†], M. Florencia Martini [1,3], María L. Cuestas [2], Verónica L. Mathet [2], Graciela Y. Moltrasio [4] and Albertina G. Moglioni [1,3,*]

[1] Cátedra de Química Medicinal, Facultad de Farmacia y Bioquímica, Universidad de Buenos Aires, Ciudad Autónoma de Buenos Aires C1113AAD, Argentina; matgmz@gmail.com (M.E.G.); flormartini1@gmail.com (M.F.M.);

[2] Instituto de Investigaciones en Microbiología y Parasitología Médica, CONICET-Universidad de Buenos Aires, Paraguay 2155, Ciudad Autónoma de Buenos Aires C1121ABF, Argentina; emilianogentile@gmail.com (E.A.G.); marilu_cuestas@yahoo.com.ar (M.L.C); vmathet@gmail.com (V.L.M.)

[3] Instituto de la Química y Metabolismo del Fármaco, CONICET-Universidad de Buenos Aires, Ciudad Autónoma de Buenos Aires C1113AAD, Argentina

[4] Cátedra de Química Orgánica II, Facultad de Farmacia y Bioquímica, Universidad de Buenos Aires, Junín 956, Ciudad Autónoma de Buenos Aires C1113AAD, Argentina; gmoltra@gmail.com (G.Y.M.)

* Correspondence: amoglio@ffyb.uba.ar or bmoglio2015@gmail.com; Tel.: +54-11-5287-4852

† These authors contributed equally to this work.

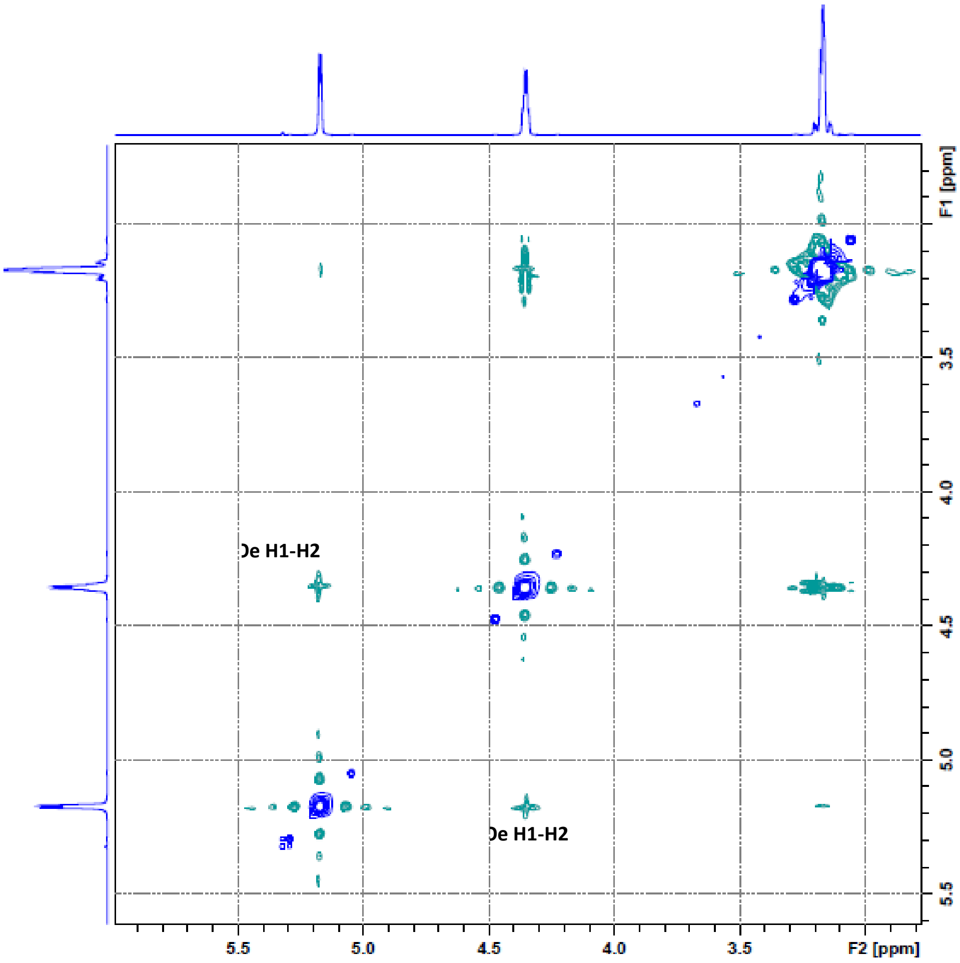

Figure S1. NOESY spectrum of compound **2.** The nOe effect between H1 and H2 show the relative configuration *cis* of substituents on positions 1 and 2.

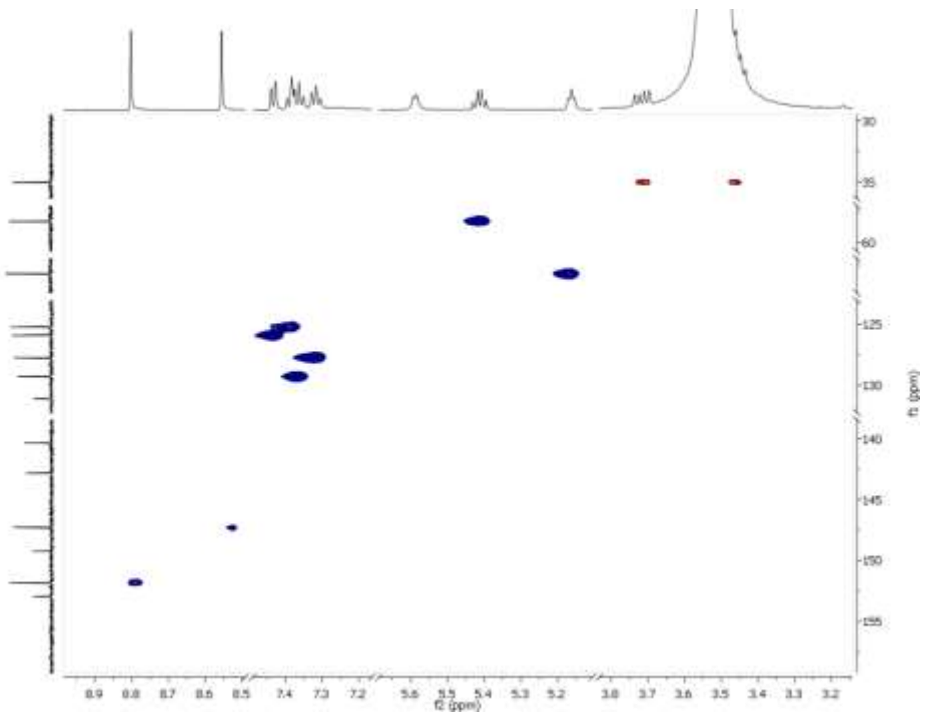

Figure S2. HSQC of compound **5**.

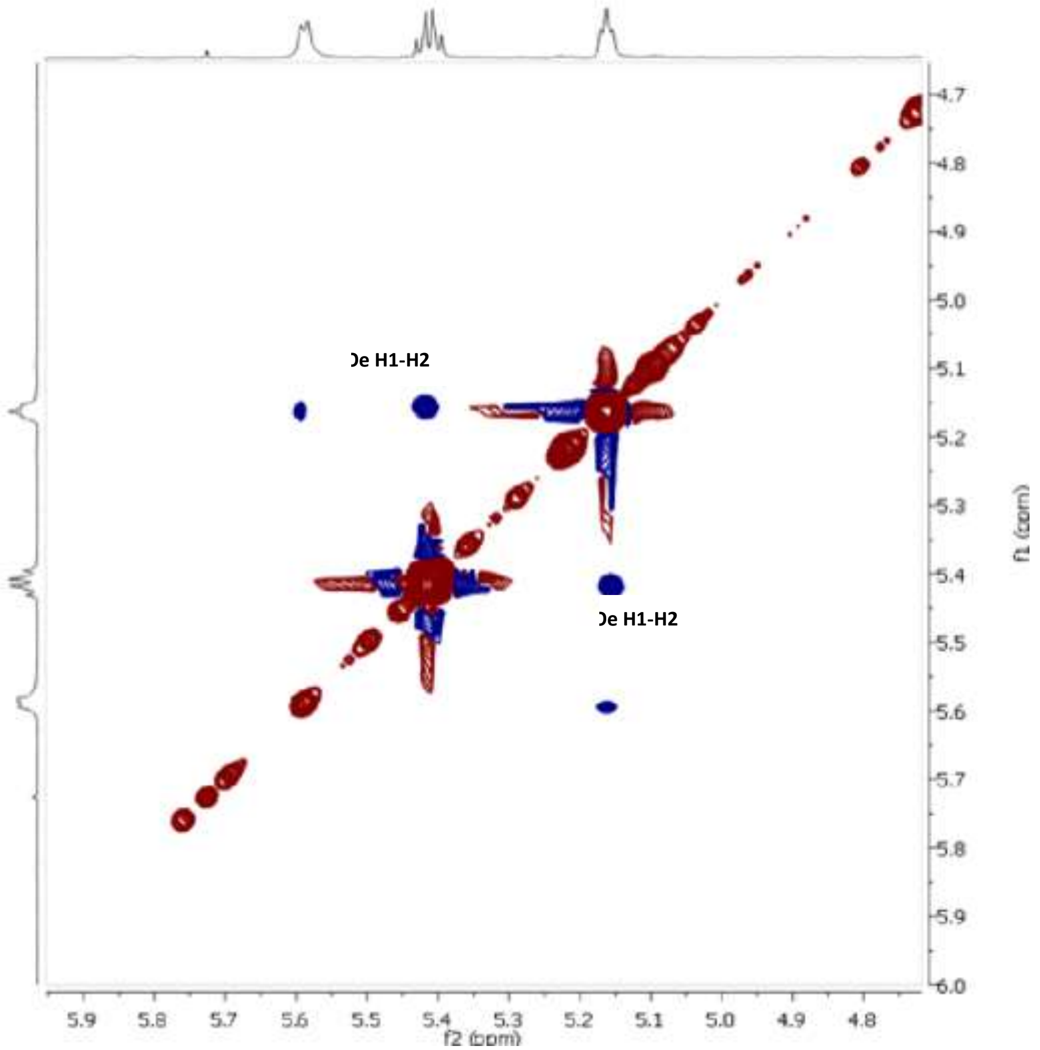

Figure S3. NOESY spectrum of compound **5.** The nOe effect between H1 and H2 show the relative configuration *cis* of substituents on positions 1 and 2.

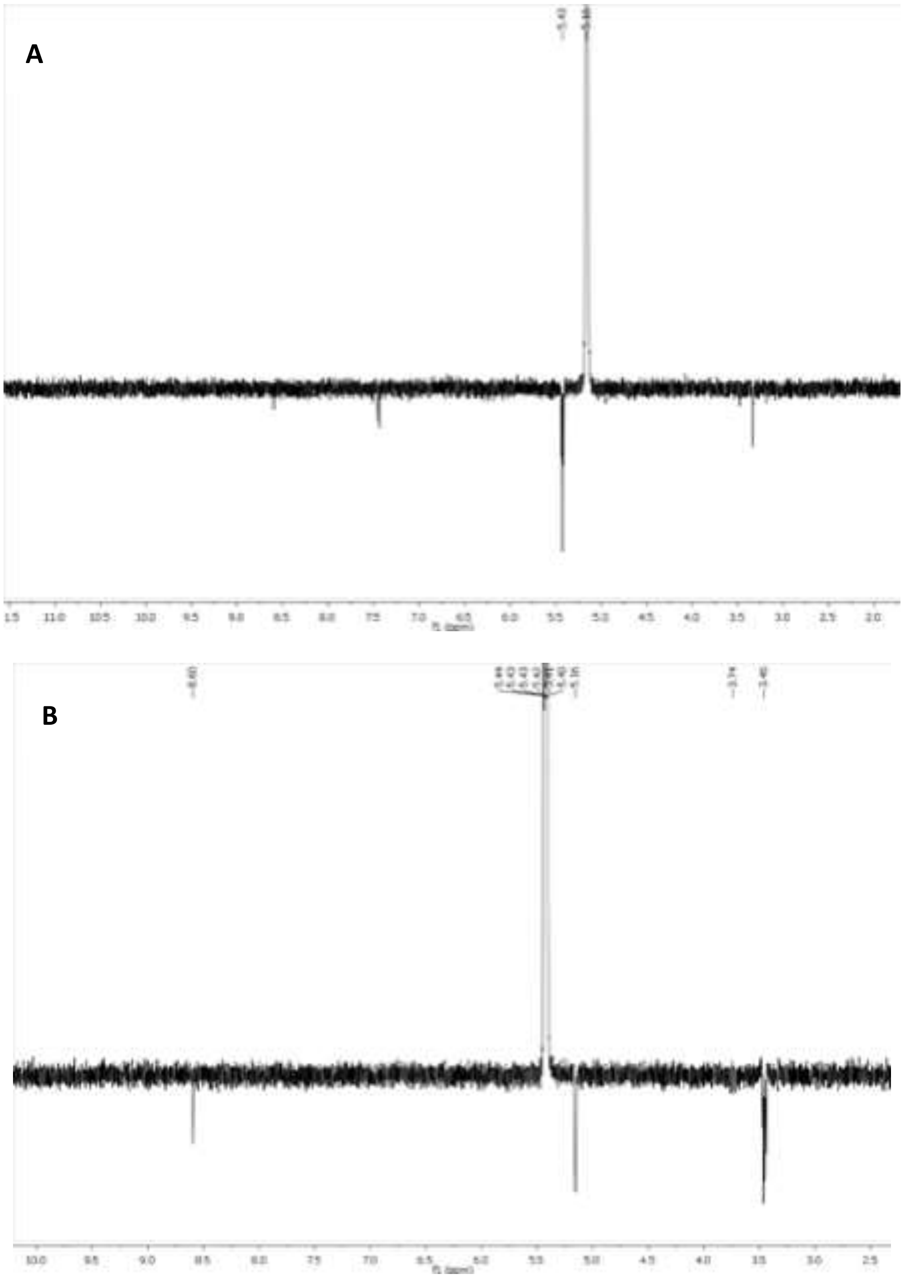

Figure S4. Selective nOe of compound **5**. A) Observed nOe when H1 was irradiated, B) Observed nOe when H2 was irradiated.
